# Reported Case of Epulis

**Published:** 1851-01

**Authors:** R. McLimont


### ARTICLE XI.

*Reported Case of Epulis.* By R. McLimont, M. D., D. D. S.

The following case happened in my practice the other day; if you think it of any interest, you can make use of it.

Mrs. S——, the wife of a laboring man in this city, called upon me a short time ago, at the advice of her medical attendant, respecting a large tumor of two years growth, situated on the upper jaw, over the incisor teeth, extending on the anterior surface from the median line to the left canine fossa, and half way across the hard palate. It was about the size of a silver dollar, had a broad base, uneven surface, hard and destitute of of sensibility. It had forced itself between the central and lateral incisors, separating these teeth about *one inch* from each other. It caused the upper lip to protrude much, and was altogether a most unsightly object. This was a very aggravated case of *epulis*, originating, in all probability, from diseased alveolar cavities, and primarily from the teeth, which were all carious and quite loose. I advised their extraction, and the removal of the tumor. I took her to Dr. D——, of this city, who fully corroborated my opinion, and appointed a day in the following week for the excision of the tumor. I was permitted to be present on the occasion. The incisions were made with a scalpel, and the bone removed with Liston's excising scissors. The cavities appeared to be diseased, but the body of the bone was sound. The tumor was exceedingly vascular and abundantly supplied with vessels. Of course the operation was followed by a considerable disfigurement and loss of intonation, both of which evils I shall try to remedy by the application of an artificial gum and palate, with teeth.

